# Impact of gut colonization by antibiotic-resistant bacteria on the outcomes of autologous stem cell transplantation in multiple myeloma

**DOI:** 10.1038/s41598-024-82589-z

**Published:** 2024-12-28

**Authors:** Marcin Jasiński, Jarosław Biliński, Martyna Maciejewska, Karolina Ostrowska, Patrycja Rusicka-Krzewska, Wojciech Konarski, Edyta Podsiadły, Emilian Snarski, Grzegorz W. Basak

**Affiliations:** 1 Department of Hematology, Transplantation and Internal Medicine, Medical University of Warsaw, Warsaw, Poland; 2 Doctoral School, Medical University of Warsaw, Warsaw, 02-091 Poland; 3 Human Biome Institute, Gdansk, 80-137 Poland; 4 Laboratory of Microbiology, University Center of Laboratory Medicine, 1a Banacha Str, Warsaw, 02-097 Poland; 5 Department of Orthopaedic Surgery, Ciechanów Hospital, Ciechanów, 06-400 Poland; 6 Department of Dental Microbiology, Medical University of Warsaw, Warsaw, 02-091 Poland; 7 Institute of Medical Sciences, University of Zielona Góra, Zielona Góra, Poland

**Keywords:** Autologous hematopoietic stem cell transplantation, Antibiotic-resistant bacteria, Gut colonization, Infection rates, Fecal microbiota transplantation, Antibiotics, Cancer

## Abstract

Patients undergoing autologous stem cell transplantation (auto-SCT) face elevated risks of infections. Additionally, patients colonized in the gastrointestinal tract with antibiotic-resistant bacteria (ARB) are at higher risk of infection with ARB and other infections. Therefore, patients colonized with ARB before auto-SCT should present with an exceptionally high incidence of infections. According to current literature, ARB colonization is the surrogate marker for dysbiosis, which is known to be associated with a diagnosis of multiple myeloma (MM). Given that, this retrospective study aimed to assess the influence of ARB colonization on infection rates, hematopoiesis regeneration, mucositis, overall survival, and progression-free survival following auto-SCT in MM. Data from 138 MM patients undergoing 141 auto-SCT were analyzed, with 15% showing ARB colonization. Among colonized patients, ESBL-producing gram-negative rods predominated. Patients with gut ARB colonization had significantly higher infection rates than non-colonized individuals (52 vs. 26%, *P* = 0.02), particularly bloodstream infections (43% vs. 14%, *P* = 0.004). Colonized patients also tended to exhibit shorter survival rates although there was no statistical significance (1-year and 2-year OS; non-colonized vs. colonized; 97 and 92% vs. 90 and 86%; *p* = 0.054). Based on our results, gut colonization before auto-SCT negatively affects treatment outcomes.

## Introduction

Resistance of bacteria against antibiotics is known as a global threat, and in 2019, approximately 5 million deaths globally were attributable to that phenomenon^1^. Predictions exist that antibiotic resistance will cause more deaths than cancer soon^2^. Therefore, patients’ colonization with antibiotic-resistant bacteria (ARB) is sometimes called the state of a “ticking bomb”. Although it is not a disease per se, ARB colonization increases the risk of mortality due to infections. That is particularly worrying in immunocompromised individuals, where mortality is reaching up to 95%^3,4^.

An important reason for ARB colonization is the impairment of the gut microbiota composition, which creates niches for new bacteria. Such damage to the gut microbiota repertoire may result from the administration of antibiotics, chemotherapeutics, or immunosuppression^5^. The Bone Marrow Transplantation Units are locations where the admitted patients are frequently colonized with ARB. That is due to the profile of individuals with impaired gut microbiome, resulting from numerous antibiotics, long and numerous hospitalizations, and microbiota-damaging therapies before allogeneic hematopoietic stem cell transplantation (alloHSCT). We have previously shown that even 31% of all patients qualified for alloHSCT are colonized with ARB^3^, but there are also centers where the colonization rate approaches 70%^6^. That alone increases the risk of systemic infections and acute graft-versus-host disease (aGHVD) incidence^3^. Our team and other authors proved that the ARB colonization before alloHSCT is also associated with the significant shortening of the overall survival (OS)^3,7–9^.

Our group revealed that fecal microbiota transplantation (FMT) performed in patients with blood disorders promotes the eradication of ARB from the gastrointestinal tract^10^. Furthermore, in the recent work of Innes et al., it was shown that fecal microbiota transplantation (FMT) in ARB carriers could mitigate the adverse outcomes seen in alloHSCT recipients, such as fever and intensive care, however, without significant impact on OS^11^. The role of FMT in the treatment of aGvHD has also been documented by us and other teams^12,13^.

Plasma cell dyscrasias are a group of diseases derived from a mutated clone of plasma cells producing the monoclonal protein. The most common disease in that group, and simultaneously the second most common hematologic malignancy, is multiple myeloma (MM). It is still an incurable disease, although considerable progress in treatment has been made in recent years due to new drug development^14^. Despite that, autologous stem cell transplantation (auto-SCT) preceded by high-dose melphalan infusion is still the most common consolidation therapy for younger and fit patients^15^. Melphalan used in conditioning is frequently associated with mucositis, typically affecting the patients a few days after the procedure^16^. Frequently, not only the oral mucosa is affected (which in particularly severe cases can lead to opioid requirement and parenteral nutrition) but the intestinal mucosa as well. That can cause the disintegration of the gut epithelium and mucosa-associated lymphoid tissue (MALT) disruption, dramatically increasing the risk of gut microbes’ influx into the bloodstream, subsequent bacteriemia, and sepsis^17^. It was also shown that the gut microbiota of patients with plasma cell dyscrasias is frequently disrupted, mainly because of antibiotics frequently prescribed for these patients. Moreover, the plasma cells have numerous direct and indirect interactions with gut epithelium, shaping the gut microbiota composition^18,19^. Taking into consideration what was previously said about ARB and that patients on hematology wards are frequently being ARB-colonized, such patients are at significant risk of life-threatening infections.

Knowing that ARB colonization is the surrogate marker for gut microbiota disruption, we wanted to learn if gut ARB colonization alone influences the rate of infections and bacteremia after auto-SCT and how it affects mortality. Furthermore, the influence of ARB colonization on other events, such as regeneration of hematopoiesis, mucositis, overall survival, and progression-free survival potentially associated with that factor, was analyzed.

## Methods

### Study design

This retrospective, single-center study used clinical data on consecutive MM patients who underwent auto-SCT between August 2016 and June 2022 in the Department of Hematology, Transplantation, and Internal Medicine of the Medical University of Warsaw, Poland.

### Materials and methods

Patients’ gut colonization data were collected during a three-month period before auto-SCT. At least one positive isolate collected from a rectal swab (performed by qualified nurse) was enough to classify the patient as colonized. Moreover, results from blood, urine, and feces samples regarding the infectious sites during the three-month period after auto-SCT were included in the study. A total of 4776 clinical specimens were analyzed during the study period.

### Data collection

Patient information was collected from the laboratory records, including species of bacteria and antibiotic sensitivity patterns.

### Microbiological procedures

Bacteria were cultured using bacteriological media according to laboratory procedures. Blood cultures were performed with Plus Aerobic/F and Anaerobic/F culture bottles (Becton, Dickinson, and Company) and monitored using the Bactec FX system (Becton, Dickinson, and Company) for 7 days. Bacterial identification was performed with MALDI-TOF mass spectrometry with Microflex LT mass (Bruker, Germany) using the MBT Compass IVD software (Bruker Daltonics, Germany) according to manufacturer instructions. Antimicrobial susceptibility testing (AST) was performed with the Kirby-Bauer disk diffusion method for the following antibiotics: ertapenem, meropenem, imipenem, temocillin, vancomycin, cefoxitin and interpreted according to European Committee on Antimicrobial Susceptibility Testing guidelines (2012). Bacteremia was defined as at least one positive blood culture, irrespective of clinical symptoms. Two consecutive positive cultures were required to confirm bacteremia caused by coagulase-negative pathogens.

The ability of isolates to produce carbapenemases (MBL, KPC OXA-48) was investigated by DDST-EDTA – double-disk synergy with ethylenediaminetetraacetic acid - detected MBL and CDT – combined disk test – detected KPC. Produce OXA-48 detected using an antibiogram disc with temocillin. Rapidec Carba NP and/or the GeneXpert qualitative real-time PCR method (Cepheid, Sunnyvale, CA) were also done.

ESBL was detected with the phenotypic confirmation method – double-disk synergy test (DDST).

### Procedures and definitions

All patients had standard anti-bacterial prophylaxis with quinolones from day 0 till engraftment. Engraftment day was defined as the day when values of white blood cells (WBC) > 1000/µl and neutrophils (NEU) > 0.5 × 10^9^/L were reached for the first time and did not decrease during the two consecutive days. Neutropenic fever was defined according to ESMO guidelines as an oral temperature of > 38.3 °C or two consecutive readings of > 38.0 °C for 2 h and an absolute neutrophil count (ANC) of < 0.5 × 10^9^/L, or expected to fall below that threshold^20^. In the case of febrile episodes during the neutropenic period, patients were treated with antibiotics targeting the gut-colonizing ARB and standard, broad-spectrum antibiotics. The severity of the disease and the patient’s status were measured with HCT-CI, and the most important features before auto-SCT were listed.

ARB bacteria were defined as bacteria resistant to clinically relevant antibiotics, which mean that given antibiotic is the last from the repertoire of classical antibiotics capable of treating in a given group, or a given antibiotic is the last oral antibiotic in a given group, etc. Generally, in this work, when defining ARB, we meant primarily antibiotic resistance determined by resistance to at least 3 classes of antibiotics (MDR - multidrug resistance), mostly ESBL, VRE and CPE (CRE), and ARB colonization was stated if one of those three resistance mechanism were present.

All patients provided standard informed consent for auto-SCT, data analysis, and publication. According to the local guidelines, rectal swabs were collected as a standard practice in all hospitalized patients. According to Polish law, the approval of the institutional review board was not required because of the noninterventional nature of the study. The study was performed in accordance with the Declaration of Helsinki and approved by the internal review board of the Department of Hematology, Transplantation, and Internal Medicine of the Medical University of Warsaw.

### Statistical analysis

The 1- and 2-year overall survival of patients after auto–SCT was analyzed. Surviving patients were censored at the last follow-up examination or the date of subsequent auto-SCT.

Patients’ baseline characteristics were summarized and tested with appropriate statistical analyses (Mann-Whitney U-test for continuous variables, the Pearson chi-square test, and Fisher’s exact test for categorical variables).

Transplant-related events and outcomes were tested with Fisher’s exact test, and the log-rank test was used for survival analysis.

## Results

### Patients characteristics

**Fig. 1:**
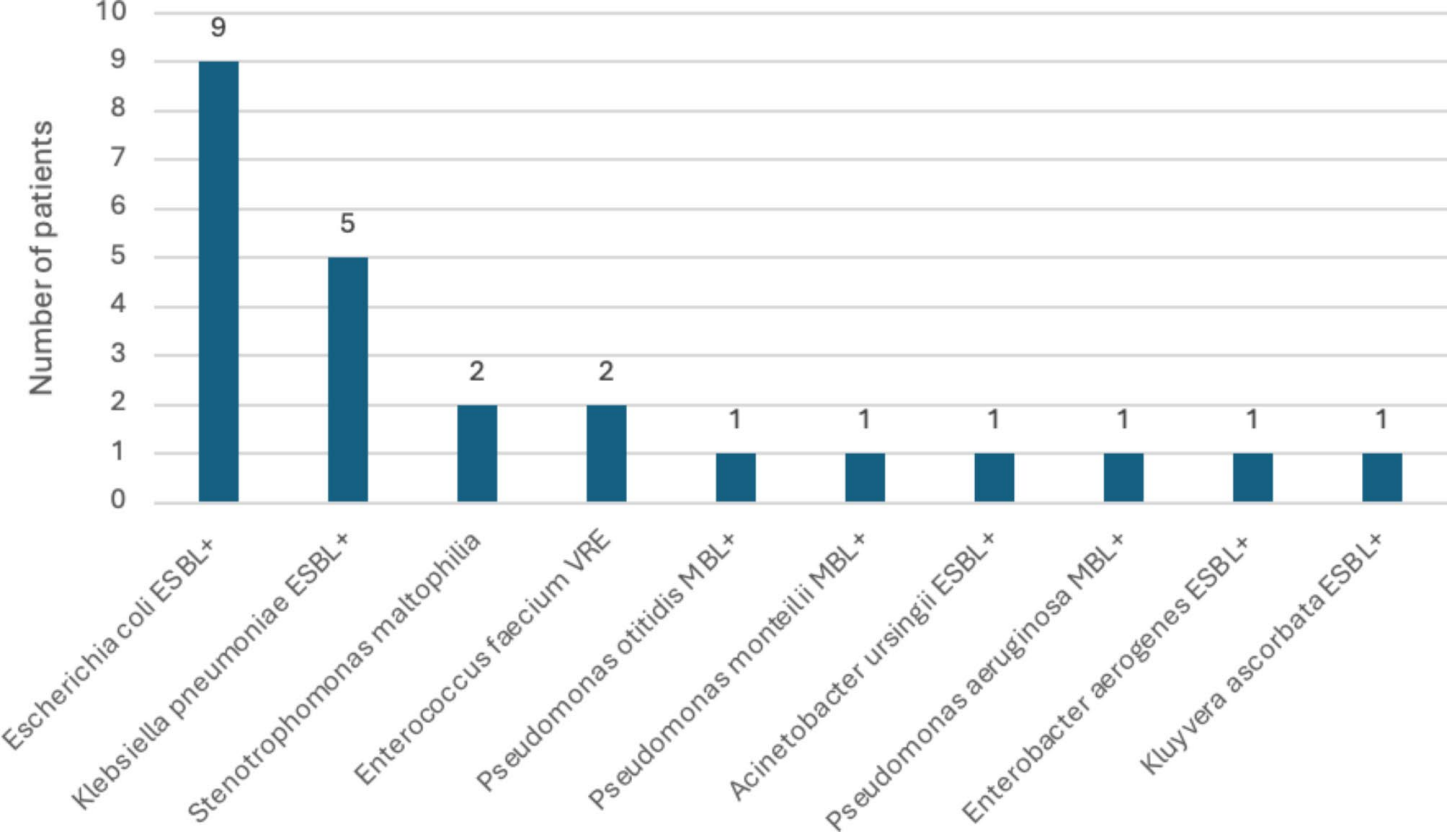
Gut-colonizing ARB pathogens in the period of 3 months before auto-SCT.

The study included 138 patients who underwent 141 auto–SCTs. Clinical characteristics and demography are shown in Table 1. There was an equal sex distribution (52% men), and the median age at the time of auto-SCT was 60 years (range, 37 to 71). The median time from the diagnosis of MM to auto–SCT was 9 months (range, 1 to 144). The most commonly administered dose of melphalan was 200 mg/m^2^ (72 auto-SCT, 51%), but the reduced dose of 140 mg/m^2^ was also common (58 auto-SCT, 41%). Most of the patients reached very good partial response (VGPR) before the auto–SCT (67 patients, 48%) or better (VGPR + CR + sCR (94 patients, 67%). At the time of auto-SCT, 15% of patients (*n* = 21) were colonized by ARB in the gut. Twenty-four ARB species were isolated from rectal swabs (3 patients had 2 colonizing ARB species). Among the most common colonizing ARB, *Escherichia coli* ESBL + was present in 6.4% and *Klebsiella pneumoniae* ESBL + in 3.5% of patients (Fig. 1). There were no significant differences in patient characteristics between groups (colonized vs. non-colonized). Most importantly, there were no differences between patients’ general statuses regarding HCT-CI.

**Table 1:** Baseline characteristics of patients included in the study.

Characteristics	All auto-SCT (*n* = 141)	Non-colonized (*n* = 120)	Colonized (*n* = 21)	*P* value
Year of autoSCT, median (range)	2018 (2016–2022)	2018 (2016–2022)	2017 (2016–2021)	NS
Male sex (N, %)	73 (52)	62 (52)	11 (52)	NS
Age at autoSCT, yr, median (range)	60 (37–71)	61 (37–71)	60 (44–70)	NS
Time from diagnosis to autoSCT, mo, median (range)	9 (1-144)	9 (1-144)	9 (5–52)	NS
Stage of disease at diagnosis (ISS) (N, %) 1 2 3	*N* = 100 31 (31) 28 (28) 41 (41)	*N* = 83 26 (31) 23 (28) 34 (41)	*N* = 17 5 (29) 5 (29) 7 (42)	NS
HCT-CI 0 1–2 3 or higher	58 (41) 54 (38) 29 (21)	53 (44) 41 (34) 26 (22)	5 (24) 13 (62) 3 (14)	0.053
Melphalan dose (N, %) 50 mg/m^2^ 100 mg/m^2^ 140 mg/m^2^ 200 mg/m^2^	*N* = 134 1 (1) 3 (2) 58 (43) 72 (54)	*N* = 113 1 (1) 2 (2) 49 (43) 61 (54)	*N* = 21 0 (0) 1 (5) 9 (43) 11 (52)	NS
Response before autoSCT (N, %) PD SD PR VGPR CR sCR	1 (1) 3 (2) 43 (30) 67 (48) 19 (13) 8 (6)	1 (1) 3 (3) 37 (30) 55 (46) 18 (15) 6 (5)	0 (0) 0 (0) 6 (29) 12 (57) 1 (5) 2 (9)	NS
Response to the therapy before autoSCT (N, %) *≤*PR *≥*VGPR	47 (33) 94 (67)	41 (34) 79 (66)	6 (29) 15 (71)	NS
Fresh/frozen cells (N, %) Fresh Frozen	*N* = 122 85 (70) 37 (30)	*N* = 104 75 (72) 29 (28)	*N* = 18 10 (56) 8 (44)	NS
Creatinine at the day of autoSCT, mmol/l, median (range)	0.94 (0.42–9.65)	0.93 (0.42–7.3)	1.27 (0.46–9.65)	NS
Isotype of IgH (N, %) IgG IgA IgD	*N* = 108 80 (63) 24 (19) 4 (3)	*N* = 93 70 (66) 20 (18) 3 (3)	*N* = 15 10 (67) 4 (27) 1 (6)	NS
LCD	58 (41)	50 (42)	8 (38)	NS
Number of cells infused (x 10^6^/kg), median, range	4.76 (2.03-27)	4.77 (2.03-27)	4.53 (3.29–9.91)	NS

LCD (light chain disease), PD (progressive disease), SD (stable disease), PR (partial response), VGPR (very good partial response), CR (complete response), sCR (stringent complete response).

### Impact of the ARB colonization on the results and complications of autoSCT

The hematopoietic cell engraftment occurred after a median of 10 days post auto-SCT (range 5–25 days). The median time of neutropenia and thrombocytopenia following the transplantation was similar between the groups. Similarly, the difference in number of days with G-CSF support was not statistically significant. Oral mucositis (OM) occurred in 60 patients (46%) overall, and the rate of OM between the groups was not statistically significant (Table 2).

**Fig. 2:**
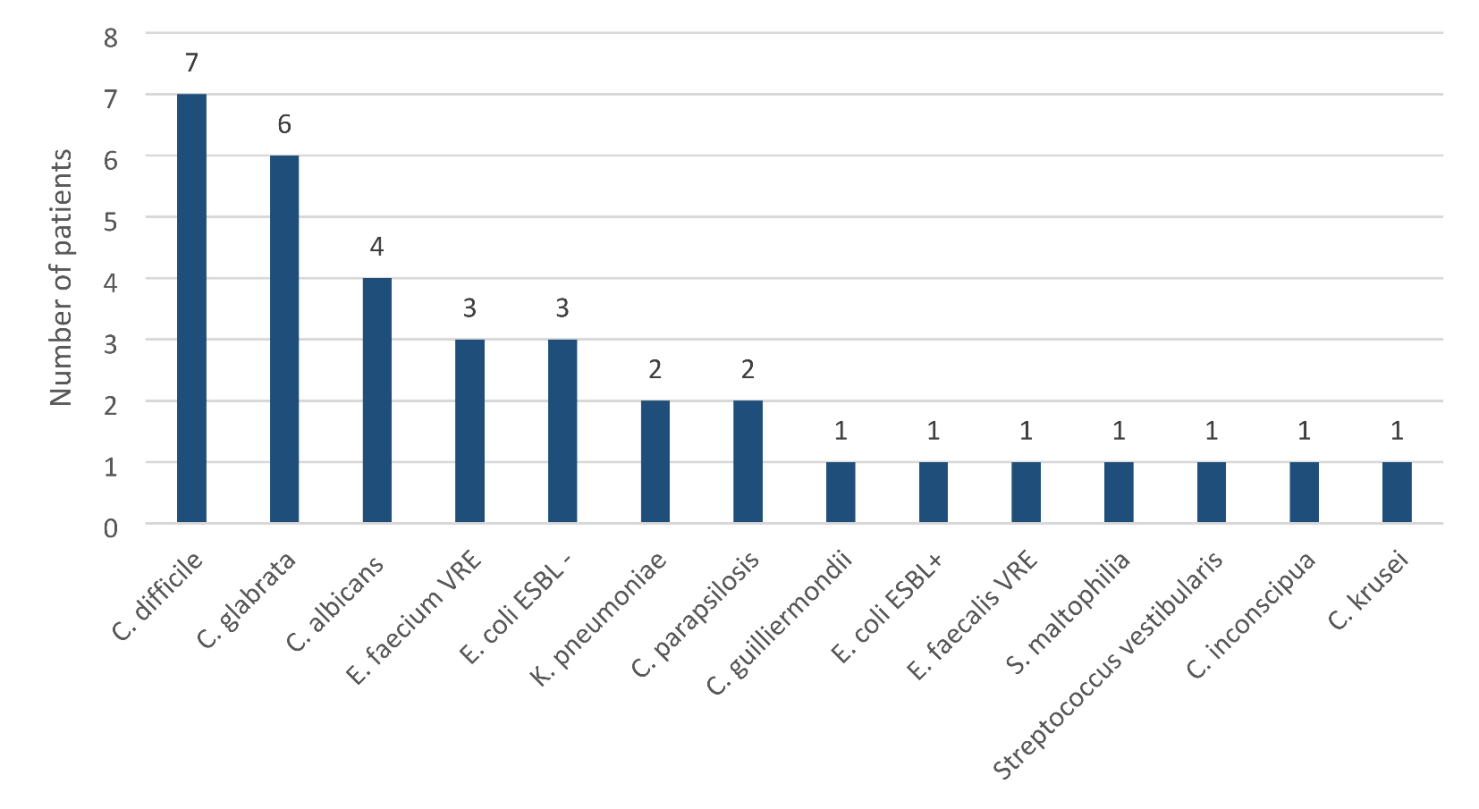
Number of patients with other than bacteriemia infections 3 months after autoSCT and their etiologies (non-colonized group).

**Fig. 3:**
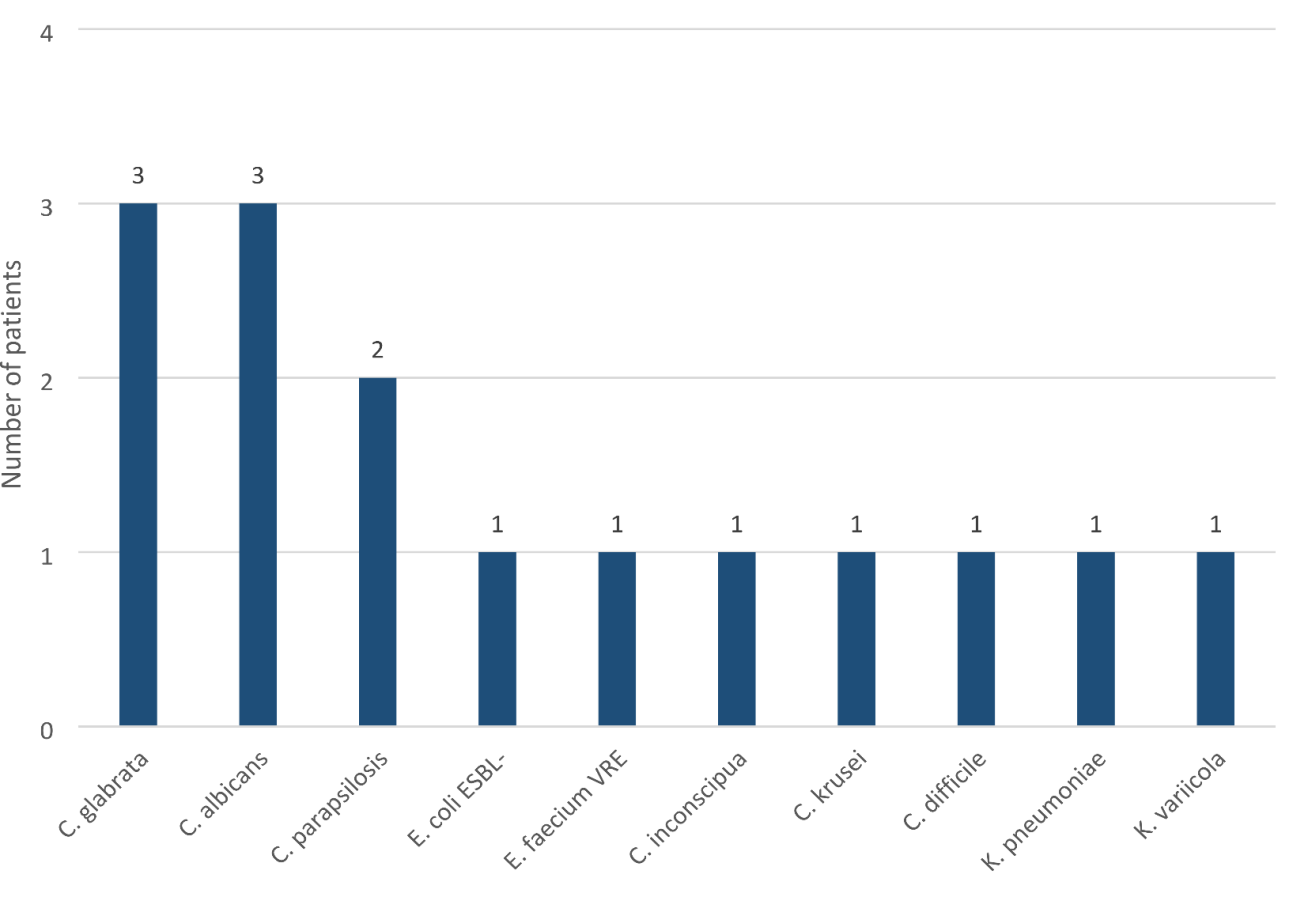
Number of patients with other than bacteriemia infections 3 months after autoSCT and their etiologies (colonized group).

**Fig. 4:**
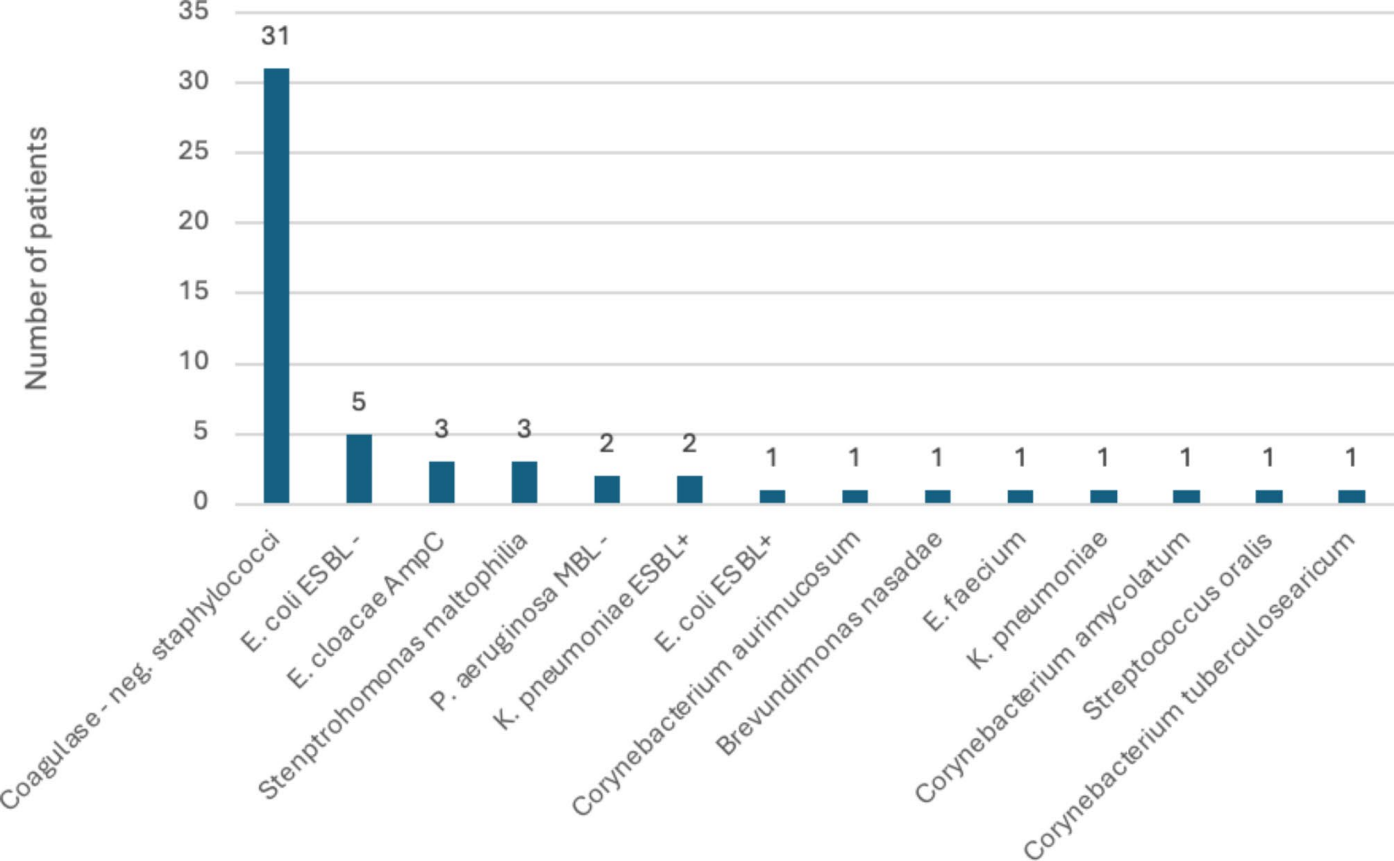
Number of patients with positive blood cultures 3 months after autoSCT and their etiologies (non-colonized group).

**Fig. 5:**
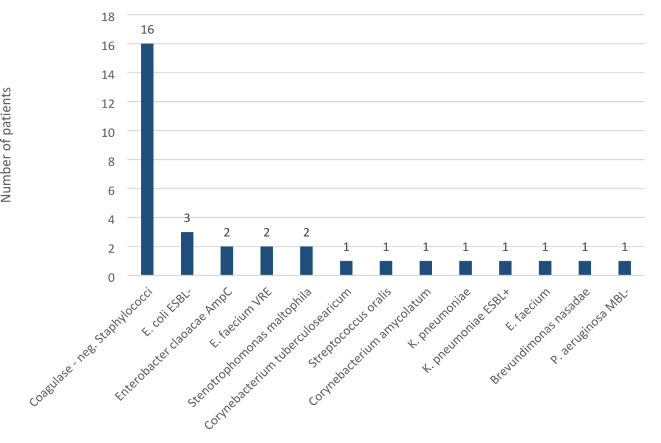
Number of patients with positive blood cultures 3 months after autoSCT and their etiologies (colonized group).

In three months following the transplantation, 30% of all patients (*n* = 42) experienced at least 1 infection. The most common etiology was *C. difficile*, responsible for pseudomembranous colitis (*N* = 6). Twenty-six (18%) had bacteriemia. Significant differences existed in the incidence of infections between the non-colonized and the gut-colonized groups (26 vs. 52%, *P* = 0.02) (Figs. 2 and 3). Moreover, there was a statistically significant difference between the non-colonized and gut-colonized groups regarding bacteremia incidence (14 vs. 43%, *P* = 0.004) (Figs. 4 and 5). What is particularly interesting, only in 2 patients in the colonized group the infection agent was the same as the gut-colonizing bacteria pre-auto-SCT (22%). In both cases, bacteriemia with a colonizing microbe was identified.

The rate of neutropenic fever in all patients was 12% (*n* = 15). The difference in neutropenic fever incidence between groups (colonized vs. non-colonized) was not statistically significant (11% vs. 20%; *p* = 0.317). The overall survival status showed no statistically significant differences between the groups. In the group of all patients, the 1-year and 2-year OS was 96 and 91%, respectively, in the median observation time of 43.0 months. There were no statistically significant differences in the case of 1-year and 2-year OS between the non-colonized vs. colonized group, respectively (97 and 92% vs. 90 and 86%; *p* = 0.054).

**Table 2:** Transplant-related events and outcomes.

Variable	All auto-SCT (*n* = 141)	Non-colonized (*n* = 120)	Colonized (*n* = 21)	*P* value
Mucositis N (%)	*N* = 130 60 (46)	*N* = 113 52 (46)	*N* = 17 8 (47)	NS
Engraftment – day; median, range	10 (5–25)	10 (5–25)	10 (9–20)	NS
Neutropenia < 1,5 G/l, median days, range	5 (0–18)	5 (0–18)	5.5 (3–18)	NS
Thrombocytopenia < 20 G/l, median days, range	12 (1–49)	12 (1–41)	12 (1–49)	NS
G-CSF, days, median, range	10 (3–23)	10 (6–23)	10.5 (3–20)	NS
Neutropenic fever N (%)	*N* = 124 15 (12)	*N* = 109 12 (11)	*N* = 15 3 (20)	NS
Patients with at least 1 infection N (%)	42 (30)	31 (26)	11 (52)	*P* = 0.02
Bacteriemia N (%)	26 (18)	17 (14)	9 (43)	*P* = 0.004

## Discussion

This retrospective, single-center analysis showed that 15% of all auto-SCT procedures involved patients being colonized with ARB. That condition influenced the infection rate – bacteriemia occurred in 43% of colonized patients, whereas it occurred only in 14% of non-colonized individuals (*p* = 0.004). Additionally, the rate of all infections was also higher. In the colonized group, it reached 52%, while in the non-colonized group, it was 26% (*p* = 0.02).

The percentage of colonized patients before auto-SCT was significantly different compared to individuals undergoing alloHSCT (31%)^3^. Furthermore, the gut colonizing bacteria were different as well. Whereas in the population of patients before alloHSCT, the bacteria species were predominantly VRE and then ESBL-producing gram-negative rods, in our cohort, the main bacteria were the latter ones and *Stenotrophomonas maltophilia* (natural resistance against carbapenems and aminoglycosides). VRE was detected only in a minimal number of patients (*n* = 2). Given that ARB colonization is the surrogate marker for poor gut microbiota repertoire (dysbiosis), it can be concluded that the impairment of gut microbiota composition is far more noticeable in the population of patients treated with alloHSCT than in the auto-SCT cohort. The reason may be the higher overall incidence of infections before alloHSCT and the more liberal usage of broad-spectrum antibiotics affecting the gut microbes (different than in an auto-SCT setting). Similar outcomes were shown in other publications, which showed the influence of ARB colonization on stem cell transplantation. In the paper published by Forcina et al., the multidrug-resistant gram-negative bacteria (MDR) colonized 16,9% and 9,6% of patients before allogeneic and autologous hematopoietic stem cell transplantation, respectively^21^.

As mentioned before, patients with MM are particularly susceptible to infections. Given the fact that the time from the first symptoms till the diagnosis of malignancy is usually longer (in contrast to acute myeloid/lymphoblastic leukemia), they are typically treated with numerous antibiotics during that time. Despite that, in our and other works, the colonization rate before auto-SCT proved much lower than in alloHSCT recipients. That may be because the time from the start of MM treatment to auto-SCT is relatively short (median 9 months; range 1-144 months), and the treatment is less intensive than in the case of acute leukemia or other aggressive cancers. In addition, contrary to individuals with acute myeloid/lymphoblastic leukemia, patients with MM are usually treated as outpatients and have a lower risk of neutropenia and associated with that infections. Given that infections remain the leading cause of mortality in patients with plasma cell dyscrasias, it is essential to decrease the colonization rate before the auto-SCT to transform the abovementioned statistics into fewer infections during the time following the procedure. One of the most promising procedures is fecal microbiota transplantation and microbiota modulation.

Analyzed data also showed no statistical differences between the groups regarding time to engraftment, days of G-CSF administration, the incidence of mucositis, days of neutropenia, or thrombocytopenia. On the contrary, it was showed that patients colonized in the gut tended to have a higher incidence of neutropenic fever following the procedure than the non-colonized individuals. However, these results proved not statistically significant (11 vs. 20%, *P* = 0.317). As previously mentioned, particularly interesting results were seen in the case of infection rates in different groups. In the whole cohort, the infection rate was 30%, whereas 18% of patients had bacteriemia. That is far less than in the alloHSCT population, which had bacteriemia in 32% of all patients, citing our previous work^3^. The most frequent bloodstream infection agent was coagulase-negative staphylococci, which is common in patients undergoing autoSCT with central venous access. In the group with ARB gut colonization, 52% of patients had at least one episode of infection during the time of 3 months after the procedure, whereas in the non-colonized group, 26% (*P* = 0.02). That shows the dominant role of gut colonization as the risk of infections after the procedure. The positive blood culture rate post-auto-SCT period showed even more interesting results. In the gut-colonized group, 43% had at least one episode of bacteremia, whereas in the non-colonized group, only 14% (*P* = 0.004). Nevertheless, when the etiology of this bacteriemia was analyzed, only in 2 patients did the etiology factor correspond with ARB colonization before auto-SCT. It is considered as ARB colonization is a marker of dysbiosis, and this increases the rate of bacterial translocation, but ARB are not “in advantage” to be translocated – it is the result of biology and statistics. Most often, the translocating bacteria is “typical gut bacteria”. The same observation was seen in our previous study with patients undergoing alloSCT^3^.

Regarding OS analysis (1-year and 2-year OS; non-colonized vs. colonized group; 97 and 92% vs. 90 and 86%; *p* = 0.054), the difference was not statistically significant, although the tendency for shorter survival in the colonized group is visible.

Previous works on ARB colonization and its effects on the auto-SCT setting mainly addressed the disruption of the gut microbiota. The group of Peled et al. showed that the gut microbiota of auto-SCT recipients is significantly injured, which is associated with increased non-relapse mortality after the procedure^22^. Joks et al. presented a study assessing the impact of multidrug-resistant gram-negative bacteria (MDRG) colonization prior to transplant on auto-SCT results in lymphoma patients. They proved that colonization with MDRG is a risk factor for septic shock development^23^.

In the previously mentioned work of Forcina et al., the MDR gram-negative bacteria colonizing the gut before auto-SCT did not significantly influence OS, transplant-related mortality (TRM), or infections-related mortality. The authors stated that mortality due to infections associated with MDR bacteria can be mitigated with pre-emptive antimicrobial treatment in cases of neutropenic fever^21^.

In other work of Scheich et al., the opposite outcomes were noted. The OS of patients undergoing autoSCT and being colonized with MDR bacteria before the procedure was significantly shorter than in the non-colonized group. That was shown in univariate (61.7% versus 73.3%, *P* = 0.005) and multivariate analysis (hazard ratio, 2.463; 95% confidence interval, 1.311 to 4.626; *P* = 0.005). Altogether, 21.7% of patients were colonized with MDR bacteria before the transplantation. Worth mentioning is the fact that not multiple myeloma (43.5%) but lymphoma (53.3%) was the most common indication for auto-SCT, which constitutes a significant difference compared with our study^24^.

Khan et al. recently showed a paper showing that patients undergoing auto-SCT had a loss of diversity of the gut microbiota after the procedure. Those patients with above-median peri engraftment diversity of fecal samples had decreased risk of progression (PFS hazard ratio, 0.46; 95% confidence interval, 0.26–0.82; *P* = 0.008) even when adjusted for disease and its status. Additionally, when fecal diversity was shown as a log_10_-transformed continuous variable, those patients with greater fecal diversity had a decreased risk of death (OS HR 0.5; 95% CI, 0.29–0.87; *P* = 0.014). However, after adjustment for disease status and its type, that association was not statistically significant (HR 0.58; 95% CI, 0.32–1.06; *P* = 0.079)^22^.

In another work of D’Angelo et al., they showed that lower alpha diversity at engraftment was associated with partial response rather than very good partial response or complete response (CR/VGPR vs. PR, *P* < 0.05). That result stems from the population of patients with MM undergoing the autoSCT^25^.

The limitations of our work should also be mentioned. First, the results are based on single-center, retrospective data. Second, groups are relatively small (particularly colonized group), and therefore, plausible conclusions cannot always be drawn. Third, the observation time of patients is relatively short, which does not allow us to draw any conclusions about the influence of gut colonization on survival time.

## Conclusions

To sum up, our work shows that the ARB colonization in the gut three months before the auto-SCT transforms into a higher rate of all infections three months after the procedure. That was particularly emphasized in terms of bloodstream infections. Considering other similar works, it is warranted that actions be taken to reverse the gut colonization of patients before auto-SCT should be taken. One emerging treatment that can potentially restore gut microbiota diversity is FMT and microbiota-based restoration therapies.

## Data Availability

No datasets were generated or analysed during the current study.
